# How Many Genetic Variants Remain to Be Discovered?

**DOI:** 10.1371/journal.pone.0007969

**Published:** 2009-12-02

**Authors:** Yudi Pawitan, Ku Chee Seng, Patrik K. E. Magnusson

**Affiliations:** 1 Department of Medical Epidemiology and Biostatistics, Karolinska Institutet, Stockholm, Sweden; 2 Center for Molecular Epidemiology, National University of Singapore, Singapore, Singapore; Baylor College of Medicine, United States of America

## Abstract

A great majority of genetic markers discovered in recent genome-wide association studies have small effect sizes, and they explain only a small fraction of the genetic contribution to the diseases. How many more variants can we expect to discover and what study sizes are needed? We derive the connection between the cumulative risk of the SNP variants to the latent genetic risk model and heritability of the disease. We determine the sample size required for case-control studies in order to achieve a certain expected number of discoveries in a collection of most significant SNPs. Assuming similar allele frequencies and effect sizes of the currently validated SNPs, complex phenotypes such as type-2 diabetes would need approximately 800 variants to explain its 40% heritability. Much smaller numbers of variants are needed if we assume rare-variants but higher penetrance models. We estimate that up to 50,000 cases and an equal number of controls are needed to discover 800 common low-penetrant variants among the top 5000 SNPs. Under common and rare low-penetrance models, the very large studies required to discover the numerous variants are probably at the limit of practical feasibility. Under rare-variant with medium- to high-penetrance models (odds-ratios between 1.6 and 4.0), studies comparable in size to many existing studies are adequate provided the genotyping technology can interrogate more and rarer variants.

## Introduction

The advent of affordable high-throughput genotyping technology has led to numerous large-scale genome-wide association studies. A striking and disappointing feature of the discoveries made is the mostly small effect sizes. The first major results in type-2 diabetes [1] reported 9 validated SNPs, one on the TCF7L2 gene having odds ratio (OR) 1.37, while the others had ORs between 1.12 and 1.20. A co-dominant model is commonly assumed, and the stated OR is per risk allele; we adopt the same model throughout. A more recent meta-analysis aiming at expanding the number of associated SNPs for type-2 diabetes [2] combined data from 3 major studies, involving 4,549 cases and 5,579 controls, using genome-wide scans of 2.2 million typed and imputed SNPs. The study identified 11 SNPs that were validated in stage 2 (21,461 subjects) and stage 3 (32,514 subjects). The ORs of these SNPs based on the combined data range from 1.05 to 1.15.

Similar results have been reported in other complex diseases: for example, in breast cancer [3] the top 11 SNPs at final validation stage (involving 21,860 cases and 22,578 controls) have ORs ranging from 1.04 to 1.26. To get an overview, we downloaded the compilation of all GWAS results from http://www.genome.gov/26525384. As of 3 March 2009 the website includes 273 publications and 1213 SNPs. We removed studies (i) of non-disease traits; (ii) that did not have replications; (iii) that did not report risk allele frequencies or p-values or ORs. ORs were computed from the largest available data, i.e. including data from the replication studies. Following (iii), quantitative traits were excluded because they did not report ORs. Using these criterion we ended up with 383 SNPs from 101 studies; the list is given in the Supplementary Material (Table S1).

**Figure 1:**
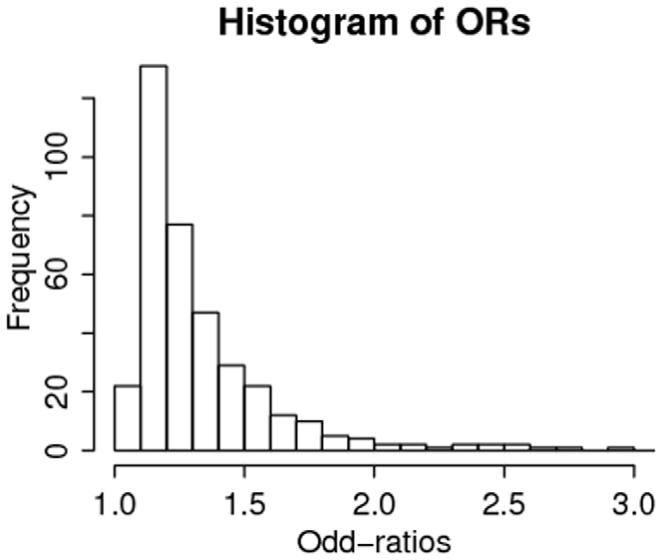
Distribution of 383 ORs from 101 GWA studies listed in the Supplementary table (Table S1).

The histogram in Figure 1 confirms that the great majority of discovered SNPs have small ORs. The median OR is 1.25. Forty percent (153/383) of the ORs are 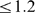; 60% (230/383) are 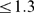 and 80% (306/383) are 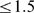. Only three percent of the ORs (10/383) are 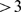. The small frequency of ORs between 1 and 1.1 suggests that many existing studies are not large enough to discover ORs in that range, and there are likely many more SNPs with ORs in that range that remain to be discovered.

As has been commented by many authors [4], [5], [6], these small effects mean that the current discoveries explain only a small fraction of the genetic contribution to the disease. In this paper we will address two questions: how many more disease-associated SNPs remain to be discovered? What sample sizes are required to discover them? The first question depends on the genetic architecture (e.g., the allele frequencies and penetrance of the remaining variants) that underlies the heritability of the disease. Intuitively, given the weak effects we observe currently, a large number of variants is required to explain a heritability of approximately 40% for type-2 diabetes [7] or 30% for breast cancer [8]. An alternative suggestion is that rare variants with higher penetrance, missed by the present genotyping, explain most of the heritability. We investigate the second question by estimating the sample size required for a case control study conducted to discover the multiple variants. It is not obvious to know, for example, what is required to discover 500 common low-penetrant variants or some other number of rare variants.

## Results

As an illustrative case study, we consider results from type-2 diabetes, where we have 9 SNPs from [1] and 11 SNPs from [2]. OR estimates are taken from the largest available combined sample. The specific SNP information is given in Table 1.

**Table 1:** The top 9 SNPs from [1] (the first 9 on the first column) and 11 SNPs from [1].

SNP	Freq.	OR	SNP	Freq.	OR
Rs10811661	0.83	1.20	rs12779790	0.183	1.11
Rs4402960	0.29	1.14	rs7961581	0.269	1.09
Rs1470579	0.30	1.17	rs7578597	0.902	1.15
Rs7754840	0.31	1.12	rs4607103	0.761	1.09
Rs1111875	0.53	1.13	rs10923931	0.106	1.13
rs13266634	0.65	1.12	rs1153188	0.733	1.08
Rs7903146	0.26	1.37	rs17036101	0.927	1.15
rs5219	0.47	1.14	rs2641348	0.107	1.10
Rs1801282	0.86	1.14	rs9472138	0.282	1.06
rs864745	0.501	1.10	rs10490072	0.724	1.05

‘Freq.’ refers to the frequency and ‘OR’ the odds-ratio of the risk allele.

**Figure 2:**
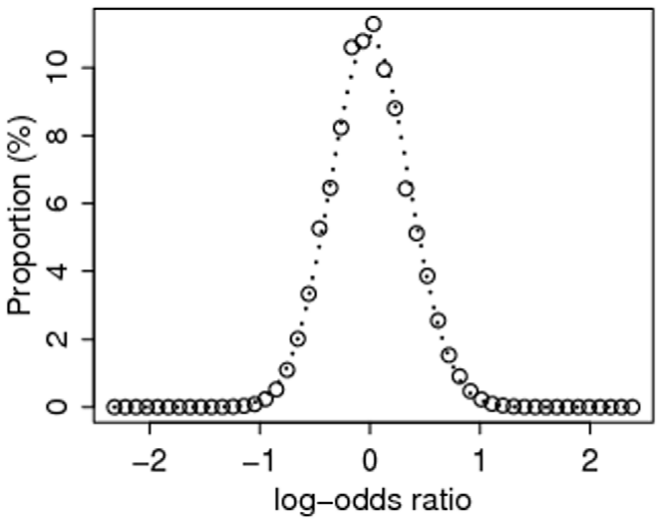
Distribution of latent genetic risk derived for the type-2 diabetes example, computed using (1) and (2).

The distribution of the risk for type-2 diabetes attributable to the SNPs in Table 1 is given in Figure 2. The sum of the proportions is 100%. The dotted curve is the normal approximation. The graph shows the population heterogeneity in susceptibility to diabetes, as assumed in quantitative genetic analysis (see Materials and Methods). Assuming 10% overall prevalence of type-2 diabetes in the population, the 5% of the population at highest risk have 16% chance of being affected. This same group has an OR of 2.1 relative to the average risk group, and an OR of 4.2 relative to the 5% at lowest risk. The great promise of genomic medicine is individualized prognosis; to achieve 90% sensitivity and 90% specificity for such a prognosis, we would need an OR of (0.9/0.1)/(0.1/0.9) = 81. This means that the current result is still very far from the goal of individualized prognosis.

The variance of the distribution in Figure 2, reflecting the contribution of the 20 SNPs, is 0.13. Using formula (4), the contribution of the 20 SNPs to the liability variance is 3.8% 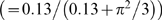. Compared to the known heritability of type-2 diabetes, which is around 40% [7], this means we have discovered only a small fraction of the potential genetic contribution to the disease. (Poulsen et al. [7] actually reported a wide range of heritability estimates (26% to 61%) depending how type-2 diabetes is defined; we take 40% as an intermediate value.) From (4), to achieve a heritability of 40%, we need 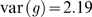, which can be achieved by discovering more variants.

### How Can We Improve the Current Results?

#### Discoveries of More Variants by Performing Larger and Larger Studies

Suppose we double the number of type-2 diabetes SNPs from 20 to 40, all assumed to be independent and of similar effect sizes to the current SNPs. Then the 5% of individuals with the highest risk have an OR of 2.7 relative to the average risk group, and an OR of 7.2 relative to the 5% at lowest risk. With 100 independent SNPs, these ORs increase to 4.5 and 20.5. However, larger studies tend to discover smaller effect sizes; beside the direct impact of increased power to detect weaker effect sizes, larger studies also increase disease heterogeneity. Distinct disease subtypes might be due to different risk alleles, so mixing all different subtypes in a large study will tend to dilute the effect sizes.

### Discoveries of Variants with Larger Effect Sizes

If we find 20 SNPs with twice the observed effect sizes in the diabetes study, the odds-ratio of the 5% at the highest risk relative to the average risk group is 4.1, and relative to the 5% at the lowest risk is 17.0. We can search for larger effect sizes by studying more homogeneous sub-populations, for example, those defined by (i) more specific phenotypes (e.g. early onset cases), or (ii) familial cases of the disease. We might also search for larger effects among gene-gene or gene-environmental interactions, where by ‘interaction’ we mean the deviation from the log-additive model.

However, studying a more homogenous sub-population will require (i) even larger overall sample sizes to overcome increased multiplicity and stratification, and (ii) more detailed data on phenotype, lifestyle and environmental factors. To illustrate the problem in the analyses of interactions, if we start with 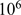 markers, just for two-way SNP-SNP interactions, we would need to search among 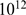 hypotheses, so severe constraints are needed to make the search practically feasible and statistically meaningful.

It is worth noting that gene-gene interactions, which are clearly plausible, also tend to generate rare composite-genotypes. Two relatively common SNPs, each with a MAF of 0.3, can for example produce an interacting genotype with a MAF of 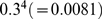, assuming independence and interaction among the minor homozygous alleles only. The problem is worse if several SNPs are interacting. So the detection of gene-gene interactions will be at least as hard as detection of rare variants.

Different types of variants, for example copy-number variants, insertion/deletion or epigenetic changes as measured by methylation status, have the potential for enlarging the search space for disease-causing variants. To contribute beyond what is already captured by the SNP array platforms, these variants have to be independent (not in linkage disequilibrium) with the existing SNP markers.

### How Many More Disease Variants Can We Discover?

The number of variants to be discovered is determined by (i) the total genetic contribution to the disease, and (ii) the genetic ‘architecture’ of the disease. This architecture is a function of the allele frequencies and effect sizes; for example, we might have common low-penetrant variants or rare high-penetrant variants. Given the current bias in genotyping common SNPs, it seems unlikely that we have missed many common-variants with medium or high effect-sizes, as they would have been discovered in the large-sample studies. What is more likely to remain are the common variants of low effect-sizes, or rare variants with low, medium or high effect-sizes.

**Figure 3:**
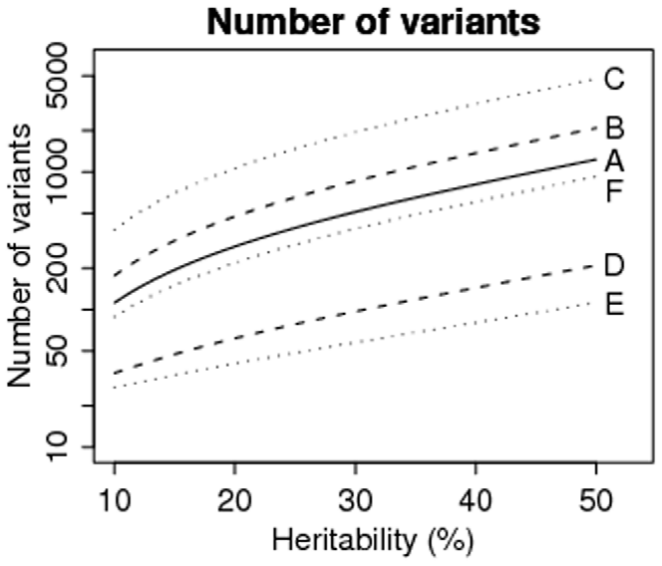
The number of variants required to explain the corresponding heritability. The labels A–F refer to the genetic models given in Table 2.

Let us first assume that the causal variants to be discovered are similar in ORs and allele frequencies to the SNPs found in [2]. Note that the OR range (1.05 to 1.15) in [2] is already lower than the range from an earlier study (1.12 to 1.20, excluding the TCF7L2, from [1]). To explain the 40% heritability of type-2 diabetes we need 812 variants (including the 20 variants already discovered). Figure 3 (solid curve) shows the number of causal variants as a function of the heritability for the common-variant model with low effect-sizes.

To get a better understanding we compare several genetic models with various distributions of MAFs and effect sizes as follows, with details given in Table 2:

- Common-low: this is as described above paragraph.
- Modest-low: the MAFs are half of the MAFs in A, but with the same effect sizes.
- Rare-low: the MAFs are one-fifth of the MAFs in A, but with the same effect sizes.
- Rare-medium: the MAFs are one-fifth of the MAFs in A, but the log-ORs are 5-times larger.
- Rare-high: the MAFs are one-tenth of the MAFs in A, but the log-ORs are 10-times larger.
- Very-rare-high: the MAFs are one-hundredth of the MAFs in A, but the log-ORs are 10-times larger.

**Table 2:** Various models of genetic architecture and the number of variants needed to explain a heritability of 0.4.

Scenario by Freq- Effect-sizes	Range of MAFs	Range of ORs	Number of variants for 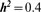
A. Common-low	0.073–0.499	1.05–1.15	812
B. Modest-low	0.0365–0.2495	1.05–1.15	1368
C. Rare-low	0.0146–0.0998	1.05–1.15	3114
D. Rare-medium	0.0146–0.0998	1.28–2.01	144
E. Rare-high	0.0073–0.0499	1.63–4.05	80
F. Very-rare-high	0.00073–0.00499	1.63–4.05	608

Figure 3 also shows the number of causal variants as a function of the heritability for the different genetic architectures. As expected, the worst in terms of potential discoveries is rare-variant low-penetrant model (C), which requires 3114 variants to explain a heritability of 0.4. In contrast, for model E, where we set the allele frequencies to be 10-times smaller (MAF range 0.0073 to 0.05) and log ORs 10-times larger (OR range 1.63 to 4.05), we only need 80 rare variants. Very-rare variants (model F, with MAF range 0.00073 to 0.005) are challenging enough just to observe them in a study, and we need approximately 600 of them even with relatively high effect-sizes. These extreme models pose other statistical difficulties, which we discuss in the next section.

### Sample Size Issues

How large should our study be to capture multiple causal variants? Most sample size computations for association studies are based on the power to detect a single variant, allowing for the standard significance level. Such an approach is not applicable to deal with the discovery of multiple causal variants, since we then have to consider the impact of multiple testing problem. We thus adapt a method from microarray gene-expression studies [9], where we consider the expected number of true positives in a list of top SNPs. Because of LD and multiple SNP markers within a haplotype block, a single causal variant may be tagged by multiple significant markers. To be concrete, assume that an average of 3 markers will be significant for each causal variant; this does not affect our conclusions. For the null SNPs, we assume that the MAFs vary according to this distribution:

MAF 0.05 0.1 0.2 0.3 0.4

proportion 0.35 0.25 0.15 0.13 0.12

This roughly follows the MAF distribution of the SNPs in chromosome 22 of the control group in the Wellcome-Trust case-control consortium data [10]. The exact shape is not crucial for our computations.

For the causal variants, under each model, the MAFs and ORs follow the distributions in Table 2. For each model, the MAFs are discretized into 5 equally-spaced values within the assumed range, and each MAF has equal proportion. The OR range is similarly split. For example, for model A, the MAFs are distributed with equal proportion at (0.073, 0.180, 0.286, 0.393, 0.500), the ORs are (1.15, 1.125, 1.100, 1.075, 1.05).

We assume that we use arrays with 1 M markers for models A and B. To be able to capture rare variants, we assume 10 M-marker arrays for model D, and 100M-marker arrays for model E.

**Figure 4:**
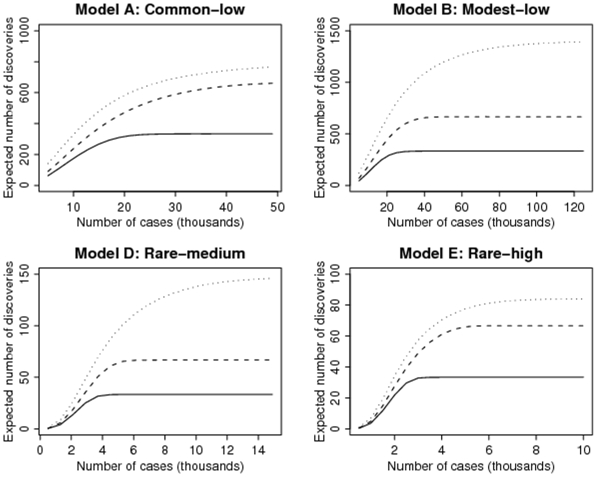
The expected number of discoveries of causal variants as a function of the number of cases in a case-control study, with equal number of controls. The models refer to those in Table 2 in terms of the range of MAFs and ORs of the risk alleles of non-null variants. For models A and B, we plot the expected number of discoveries among the top 1000 (solid), 2000 (dashed) and 5000 SNPs (dotted); for models D and E, they among the top 100 (solid), 200 (dashed) and 500 SNPs (dotted).

Figure 4 shows the expected number of causal variants that will be discovered as a function of the number of cases in a case-control study, with equal number of controls. For example, in model A, to capture about 330 of the 812 causal variants in the top 1000 SNPs, we need a study with at least 25,000 cases and 25,000 controls. (Since we assume 3 significant markers per causal variant, when all the causal variants are discovered with large enough sample size, the top 1000 SNPs will in the average contain the top 333 causal SNPs.) In such a study, we expect about 600 of the 812 causal variants in the top 5000 SNPs. The large number of null SNPs in this list of top 5000 SNPs means that further validation studies are required to identify the causal variants. Approximately 50,000 cases and 50,000 controls are needed to capture the 812 causal variants among the top 5000 SNPs.

The worse scenario regarding sample size is model B, the model of rare variants with low effect-sizes, where at least 125,000 cases and 125,000 controls are needed to discover approximately 1400 variants among the top 5000 SNPs. However, the detection of rare variants in models D and E is surprisingly within reach with the kind of sample sizes achieved by consortium studies performed today. This is of course a function of the assumed MAFs and ORs; if we reduce the MAFs or the ORs, or both, the sample size requirement will increase.

## Discussion

Our current search in genome-wide association studies (GWAS) is based on the common-disease common-variant model. It might be argued that the distribution of validated SNPs supports this model [5]; for example, 18 of the 20 validated SNPs for type-2 diabetes in Table 1 have MAFs 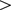
10%. Of the 383 SNPs from the recent GWAS (see Introduction), 87% (335/383) have MAFs 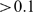. This observation is of course biased since statistical power is higher for larger MAFs and the current genotyping technology prioritizes SNPs with larger MAFs. The current array technology from Affymetrix and Illumina, directly and indirectly via LD, has a good coverage of the HapMap 4 M SNPs. However, an assessment in a resequenced region of 76 genes [11] shows that the current products, including Affymetrix 6.0 and Illumina 1 M, have substantially low coverage of the complete common variation with MAFs 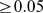. So there could still be other common causal variants that are not yet covered by existing arrays.

We have used heritability as the basis to estimate the number of remaining variants, where heritability is defined as the genetic contribution to the variance of the liability of the disease. In comparison, Yang et al. [12] used the population attribution fraction (PAF), roughly the genetic contribution to the proportion of the disease in the population. While it is straightforward to compute the PAF from a set of known SNPs, it is not obvious how to get the total PAF from all the (known and unknown) causal variants. This is a disadvantage compared to our approach, since heritability is commonly reported for most diseases.

Our computation shows that a large number of low-penetrant variants are needed to account for a heritability of 30–40%. This poses a major challenge, requiring enormous sample sizes (e.g. model B in Figure 4 to discover these variants. While such large samples are feasible in some existing consortia, a complicating factor that comes with larger and larger studies is the potential dilution of signal that results from the need to include heterogeneous populations and/or heterogeneous phenotypes. For example, it is clear from studies on the hereditary forms of breast cancer that mutations in the BRCA1 and BRCA2 genes are often specific to individual populations [13]. If distinct sub-phenotypes are due to different susceptibility genes, a study that combines these heterogeneous phenotypes will yield diluted effects.

A smaller number of rare medium- to high-penetrant variants are needed to account for the heritability. The current SNP array platforms are not able to genotype very rare SNPs, but, surprisingly, if denser arrays were available and the ORs were of medium size (e.g, 1.28 to 2.01 in model D), we would only need modestly large sample sizes to detect these rare variants. Such sample sizes are comparable to many existing genome-wide association studies, so they are well within reach. We might also search for higher-penetrant variants in subsets of populations, for example, by more strictly-defined phenotypes or by studying familial cases.

One natural question about the rare-variant model with large effect-sizes (e.g., model E) is whether existing data already rule it out. Is it possible to miss such rare alleles using the existing tagging SNPs? The case of the CHEK2 1100delC mutation is a relevant example. It has an allele frequency of approximately 0.5% and an OR of 2.7 for sporadic breast cancer and 4.8 for familial breast cancer [14]. Yet the CHEK2 gene does not appear among the top SNPs in the largest most recent breast cancer association study [3]. So rare-variant model with large effect-sizes is still a possibility.

Very rare variants (MAFs
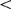
0.01) will create methodological problems. First of all, they are not represented in the current highest-density genotyping arrays. Another problem is the measurement accuracy: since genotype calling is based on fluorescent intensity and clustering, it will be hard to distinguish very rare variants from genotyping errors. Also, as they are likely to occur after the out-of-Africa migration, rare variants are likely to be population specific, which means that we cannot simply combine different study cohorts. Some of these problems might be solved by the complete sequencing method, but this technology is still too expensive for large studies.

Age-related macular degeneration [15] and exfoliation glaucoma [16] are unusual among phenotypes studied through GWAS, with large effects from common variants that have been identified in limited samples. Nonetheless, they show that there are traits with marked allelic homogeneity. Other very recent example is transferrine concentration [17], where 40% of the variance is explained by a single locus. However, it is impossible to judge beforehand which complex traits will display such a genetic architecture.

To appreciate the scope of our challenge in genetic dissection of complex phenotypes, it is useful to consider the genetics of cystic fibrosis (CF), a ‘simple’ Mendelian disease of the mucus glands of the lungs, liver and pancreas. CF is a recessive disorder, caused by mutations in CFTR, a 230,000-base long gene on chromosome 7q31.2. Deletion of codon 508 (phenylalanine), first identified in 1988 [18], is found in 66% of the cases. However, there are more than 1000 other deleterious mutations, a great majority of which are very rare variants. It is known that the clinical manifestations of the disease, for example prognosis, vary substantially; while these correlate with the type of mutations [19], [20], the genotype explains only a small portion of the clinical variability.

This highlights two salient points: (i) If a simple genetic disease such as CF can have more than 1000 functional deleterious variants, are there reasons to believe that the number and spectrum of functional mutations (in terms of non-synonymous substitutions, stop-mutations, deletions, splice mutations etc.,) should be different for genes with more subtle effects on complex diseases? (ii) Monogenic diseases such as CF also have phenotypic diversity, and this diversity is still poorly explained by the underlying genetics. If anything, the phenotypic diversity of within each complex disease tends to be wider than that of simple Mendelian diseases, so our challenge will be even greater. Different disease subtypes are likely due to different (combinations of) causal variants; however, due to sample-size problems, our case-control samples are combined over these subtypes, so, the effects of the functional variants will be diluted. In conclusion, substantial challenges remain in finding genetic explanation of the common diseases.

## Materials and Methods

### Heterogeneity in Susceptibility

In quantitative genetic analysis of a complex disease we usually assume a latent susceptibility (or liability) that varies between individuals [21]. The liability can be due to genetic and environmental factors; heritability is the proportion of the variance in liability due to genetic factors. Putting existing discoveries into this framework helps answer our questions.

Starting with the estimated odds-ratio and allele frequency for each SNP, assume that the SNPs act independently and multiplicatively. Suppose we have 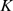 SNPs with MAF 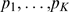. Each SNP generates 3 genotypes (AA, AB or BB) with frequencies 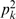, 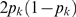 and 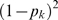. Assuming the 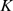 SNPs combine randomly, there are 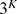 possible combinations, each with associated log OR and proportion given by:
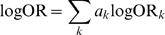
(1)

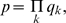
(2)where the sum and the product is over different SNPs in the configuration, 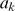 is the number of risk alleles (0, 1 or 2) and 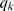 is the frequency of the 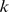
th genotype; 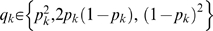 depending on the genotype.

The collection of log ORs with its proportions represents the risk distribution implied by the collection of SNPs. For 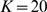, the total number of combinations 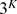 is very large, so we need to group the log ORs into intervals and combine the proportions accordingly. Such grouping is also useful for plotting; see Figure 2.

From the risk distribution we can evaluate its variance. Since each term in the summation (1) is a scaled-binomial variate with parameters 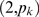 and log OR as the scale, the variance of the distribution is given by
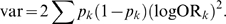
Here we see the relationship between the number of variants and the variance of the risk distribution: if we add more variants into the formula above we will increase the variance. For example, if we double the number of variants with another set that has similar MAFs and ORs, we will double the variance; i.e., the number of variants varies linearly with the variance. Thus finding the number of variants to achieve a certain variance is straightforward.

The number of variants is connected to heritability through the variance. First note that the log OR in Figure 2 corresponds directly to the latent susceptibility model well known in statistical genetics:
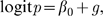
(3)with random genetic effect 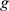 distributed as 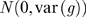 (e.g., [22]). In this model, the constant term 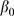 is determined the overall prevalence of the disease. The contribution of the genetic factors to the liability of the disease is so-called heritability:
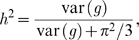
(4)where 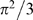 is the variance of the standard logistic distribution [22].

### Sample Size Computation

For each SNP, consider the observed log OR as the test statistic, and let 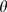 be the true OR. In a case-control study of 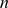 cases and 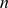 controls, the observed log OR is approximately 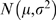, with
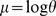
(5)

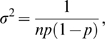
(6)where 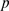 is the MAF of the SNP. The parameter 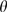 is the OR per allele, and we assume Hardy-Weinberg equilibrium so each subject contributes two independent alleles for each SNP.

To use the method in [9], we first need 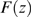, which is the marginal distribution of the statistics from all SNPs. In general, accounting the contribution from all the SNPs, 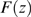 will follow a mixture distribution of the form:
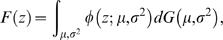
(7)where the 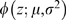 is the normal density with mean 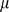 and variance 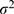, and 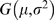 is the joint distribution of mean and variance of the log ORs across the SNPs. From (5) and (6), 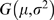 is determined by the joint distribution of true OR and MAF across the SNPs. Thus we can study the effect of various distributions of MAFs and ORs on the sample size needed to detect the non-null SNPs. From the mixture model we can also get 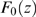, the marginal distribution of the null SNPs, those that are not associated with the case-control status.

In practice the joint distribution of ORs and MAFs is discretized, as given in the example in the Results section, so the mixture (7) becomes
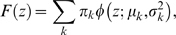
where the index 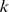 runs over all possible (OR, MAF)-combinations, and 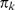, 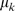 and 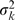 are the corresponding proportion, mean and variance associated with the 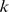
th (OR, MAF)-combination. For the null SNPs we get
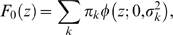
where now 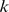 runs over all the (OR, MAF)-combinations with true OR 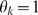. These distributions give the false discovery rate (FDR), using
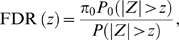
where 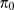 is the proportion of null SNPs, assumed very close to one, and 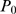 and 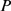 are the probabilities computed under the distributions 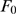 and 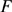, respectively. Once we have the FDR, we can use the method in [9] to evaluate the sample size required to achieve a certain FDR level. Finally, given a certain FDR level, the expected number of discoveries in a collection of 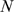 top SNPs is 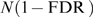.

## Supporting Information

Table S1 Table of Recent GWAS used in the Introduction
(1.01 MB XLS)

## References

[1] Diabetes Genetics Initiative (2007). Genome-wide association analysis identifies loci for type 2 diabetes and triglyceride levels.. Science.

[2] Zeggini E, Scott LJ, Saxena R, Voight BF, Marchini JL (2008). Meta-analysis of genome-wide association data and large-scale replication identifies additional susceptibility loci for type 2 diabetes.. Nat Genet.

[3] Easton DF, Pooley KA, Dunning AM, Pharoah PD, Thompson D (2007). Genome-wide association study identifies novel breast cancer susceptibility loci.. Nature.

[4] Wray NR, Goddard ME, Visscher PM (2008). Prediction of individual genetic risk of complex disease.. Curr Opin Genet Dev.

[5] Iles MM (2008). What can genome-wide association studies tell us about the genetics of common disease?. PLoS Genet.

[6] Murray SS, Schork NJ, Topol EJ, Frazer KA (2009). Human genetic variation and its contribution to complex traits.. Nat Rev Genet.

[7] Poulsen P, Kyvik KO, Vaag A, Beck-Nielsen H (1999). Heritability of type II (non-insulin-dependent) diabetes mellitus and abnormal glucose tolerance – a population-based twin study.. Diabetologia.

[8] Lichtenstein P, Holm NV, Verkasalo PK, Iliadou A, Kaprio J (2000). Environmental and heritable factors in the causation of cancer–analyses of cohorts of twins from Sweden, Denmark, and Finland.. N Engl J Med.

[9] Pawitan Y, Michiels S, Koscielny S, Gusnanto A, Ploner A (2005). False discovery rate, sensitivity and sample size for microarray studies.. Bioinformatics.

[10] Wellcome Trust Case Control Consortium (2007). Genome-wide association study of 14,000 cases of seven common diseases and 3,000 shared controls.. Nature.

[11] Bhangale TR, Rieder MJ, Nickerson DA (2008). Estimating coverage and power for genetic association studies using near-complete variation data.. Nat Genet.

[12] Yang Q, Khoury MJ, Friedman J, Little J, Flanders WD (2005). How many genes underlie the occurrence of common complex diseases in the population?. Int J Epidemiol.

[13] Ferla R, Caló V, Cascio S, Rinaldi G, Badalamenti G (2007). Founder mutations in BRCA1 and BRCA2 genes.. Ann Oncol.

[14] Weischer M, Bojesen SE, Ellervik C, Tybjaerg-Hansen A, Nordestgaard BG (2008). CHEK2*1100delC genotyping for clinical assessment of breast cancer risk: meta-analyses of 26,000 patient cases and 27,000 controls.. J Clin Oncol.

[15] Klein R, Zeiss C, Chew E, Tsai JY, Sackler RS (2005). Complement Factor H Polymorphism in Age-Related Macular Degeneration.. Science.

[16] Thorleifsson G, Magnusson KP, Sulem P, Walters GB, Gudbjartsson DF (2007). Common sequence variants in the LOXL1 gene confer susceptibility to exfoliation glaucoma.. Science.

[17] Benyamin B, McRae AF, Zhu G, Gordon S, Henders AK (2009). Variants in TF and HFE explain approximately 40% of genetic variation in serum-transferrin levels.. Am J Hum Genet.

[18] Drumm ML, Smith CL, Dean M, Cole JL, Iannuzzi MC (1988). Physical mapping of the cystic fibrosis region by pulsed-field gel electrophoresis.. Genomics.

[19] McKone EF, Emerson SS, Edwards KL, Aitken ML (2003). Effect of genotype on phenotype and mortality in cystic fibrosis: a retrospective cohort study.. Lancet.

[20] McKone EF, Goss CH, Aitken ML (2006). CFTR genotype as a predictor of prognosis in cystic fibrosis.. Chest.

[21] Sham PC (1997). Statistics in Human Genetics..

[22] Noh M, Yip B, Lee Y, Pawitan Y (2006). Multicomponent variance estimation for binary traits in family-based studies.. Genet Epidemiol.

